# Brain Activation in Primary Motor and Somatosensory Cortices during Motor Imagery Correlates with Motor Imagery Ability in Stroke Patients

**DOI:** 10.5402/2012/613595

**Published:** 2012-12-29

**Authors:** Linda Confalonieri, Giuseppe Pagnoni, Lawrence W. Barsalou, Justin Rajendra, Simon B. Eickhoff, Andrew J. Butler

**Affiliations:** ^1^Department of Human Science “Riccardo Massa”, Centre for Studies in Communication Sciences (CESCOM), University of Milan-Bicocca, 20162 Milan, Italy; ^2^Studi Cognitivi, Cognitive Psychotherapy School and Research Center, Foro Buonaparte 57, 20121 Milan, Italy; ^3^Department of Biomedical Sciences, University of Modena and Reggio Emilia, Modena, Italy; ^4^Department of Psychology, Emory University, Atlanta, GA 30322, USA; ^5^Department of Rehabilitation Medicine, School of Medicine, Emory University, Atlanta, GA 30322, USA; ^6^Cognitive Neuroscience Group, Institute of Clinical Neuroscience and Medical Psychology, Heinrich-Heine University Düsseldorf, Germany; ^7^Institut für Neurowissenschaften und Medizin, Forschungszentrum Juelich, 52428 Jülich, Germany

## Abstract

*Aims*. While studies on healthy subjects have shown a partial overlap between the motor execution and motor imagery neural circuits, few have investigated brain activity during motor imagery in stroke patients with hemiparesis. This work is aimed at examining similarities between motor imagery and execution in a group of stroke patients. *Materials and Methods*. Eleven patients were asked to perform a visuomotor tracking task by either physically or mentally tracking a sine wave force target using their thumb and index finger during fMRI scanning. MIQ-RS questionnaire has been administered. *Results and Conclusion*. Whole-brain analyses confirmed shared neural substrates between motor imagery and motor execution in bilateral premotor cortex, SMA, and in the contralesional inferior parietal lobule. Additional region of interest-based analyses revealed a negative correlation between kinaesthetic imagery ability and percentage BOLD change in areas 4p and 3a; higher imagery ability was associated with negative and lower percentage BOLD change in primary sensorimotor areas during motor imagery.

## 1. Introduction

The residual disability after stroke is substantial, with about 65% of patients at 6 months unable to effectively incorporate the paretic hand into daily activities [1, 2]. In turn, the degree of functional deficit contributes to a reduced quality of life after stroke [2–6]. Studies on healthy volunteers have shown that mental practice with motor imagery can improve the performance of motor skill behaviours [7, 8]. Motor imagery can be defined as a dynamic state during which the representation of a specific motor action is internally reactivated within working memory without any overt motor output [9]. According to the motor simulation hypothesis [10], and the grounded cognition perspective [11], represented actions correspond to covert, quasi-executed actions, involving a partial reenactment of the mechanisms that normally participate in various stages of action generation [10]. Therefore, motor imagery is a cognitive process based on sensorimotor simulation mechanisms, where individuals implicitly reenact actions without producing an overt motor output [10]. A certain degree of similarity between brain areas activated during motor imagery and execution has indeed been demonstrated [10, 12], providing empirical support for such theoretical framework.

A combination of mental practice and physical therapy is recognized as a valuable technique to facilitate patients' motor recovery and has few, if any side effects or contra-indications. A meta-analysis by Driskell and colleagues [7] indicates that mental practice is an effective means for enhancing performance, although it is by itself less effective than physical practice. In a classic study by Yue and Cole [8] healthy participants who trained mentally, displayed an improvement of 22% in muscle strength following isometric contraction of the abductor muscles of the fifth's digit metacarpophalangeal joint. This strength improvement was greater than a no-practice condition (improvement of 3.7%), but less than those who trained physically (improvement of 33%). Page and colleagues [13] showed that poststroke patients treated with a program that included both physical and mental practice exhibited significantly greater reduction of affected arm impairment as compared to those receiving physical practice and relaxation exercises. Specifically, patients receiving both physical and mental practice improved an average of 7.81 points on the Action Research Arm test (ARA) and 6.72 points on the Upper Extremity Fugl-Meyer Assessment of Motor Recovery After Stroke (FM), whereas patients subjected to physical practice and relaxation showed a significantly lower improvement of only 0.44 points on the ARA and 1 point on the FM.

The use of mental practice in poststroke rehabilitation traces its roots to the observation of an important overlap between the neural circuits activated by motor imagery and motor execution [10]. In this light, mental practice may represent a useful primer to re-activate and stimulate sensorimotor networks damaged after stroke, leading to remediation of functional impairment. However, it is presently unclear to what extent patients with motor impairment following stroke retain the ability to cognitively reenact or simulate movements of their impaired limbs [10, 14–16] and whether this depends on the partial overlap between the neural substrates of motor execution and imagery being preserved.

The similarity in the neural bases of motor imagery and execution has been empirically verified in healthy subjects. Several studies involving healthy participants have shown that premotor cortices (BA 6) and parietal areas (inferior parietal lobule) are active during both motor imagery and overt execution [17–19], while the involvement of primary motor cortex (M1) in motor imagery is less consistent. Some studies reported a lack of activation of primary motor cortex during imagery of finger movements, in contrast to actual motor execution, in healthy volunteers [18, 20]. Other studies have detected a slight increase of activity in M1 during motor imagery of sequential finger movements, albeit with a lower intensity than during real execution [21]. The notion that increased activation in M1 during motor imagery reflects mild muscular contractions has been ruled-out by studies using electromyography (EMG) monitoring during MRI scanning, as well as transcranial magnetic stimulation (TMS) [22, 23]. Interestingly, evidence provided by Solodkin and colleagues [24] (see also [21]) suggests that M1 is active during kinaesthetic imagery, but not during visual imagery. Gerardin and colleagues showed a degree of interindividual variability in the activation of primary motor cortex during motor imagery that may be related to individual differences in the ability to imagine movements and simulate kinaesthetic sensations [20].

Although there is mounting evidence for a partial overlapping of motor execution and motor imagery cortical circuits, there is currently little data about the integrity of such shared network in hemiparetic stroke patients. Kimberley and colleagues [25] examined the similarities between motor imagery and motor execution in terms of hemispheric balance in stroke patients with severe hemiplegia. These patients had no active movement in their affected hand secondary to stroke (Upper extremity Fugl-Meyer score range: 9–14). Using fMRI they demonstrated ipsilateral activation in M1 and supplementary motor area (SMA) but only contralateral activation in the primary somatosensory cortex (S1) during motor imagery and execution of a wrist-tracking task with the affected hand. Notably, the study by Kimberley et al. focused on specific, *a priori *selected ROIs, rather than investigating functional similarities between motor execution and motor imagery across the entire brain. Cortical reorganization associated with the mental practice of movement simulation has been examined in a study by Johnson-Frey [14], in a small sample of three patients with severe hemiplegia due to capsular stroke. Their study showed increased activation in parietal, motor, and supplementary motor areas contralateral to the affected limb during motor imagery, following a 9-week period of daily practice of computerized movement simulation tasks. However, the issue of whether baseline (i.e., pretraining) similarities in brain activations during motor imagery and motor execution are conserved in stroke patients, despite its relevance for mental practice interventions, has not yet been directly examined.

The present study investigates the cortical substrates of motor imagery and motor execution in a cohort of chronic stroke patients. This study aims at detecting the similarities between circuits activated by motor imagery and motor execution in stroke patients and to test for correlations between the magnitude of brain activation during motor imagery and individual differences in motor imagery ability. We hypothesized that the performance of motor imagery and motor execution involving the affected hand would show similar and partially overlapping patterns of brain activations, especially in contralesional premotor cortices (Brodmann Area 6) and parietal areas. This hypothesis is based on extant evidence of shared patterns of brain activations between motor imagery and execution in premotor and parietal areas in healthy subjects [18, 20]. Our secondary hypothesis was that brain activation in contralesional primary motor cortex (M1) and ipsilesional primary somatosensory cortex (S1) during a motor imagery task would positively correlate with motor imagery ability scores. This hypothesis is based on the notion that a greater ability in imagining kinaesthetic sensations will give rise to a more intense sensorimotor reenactment, mediated by increased activation of the relevant sensorimotor circuits [11]. As already suggested, but not empirically verified [20], we propose that individual variability in motor imagery ability modulate the involvement of primary motor and somatosensory cortex during motor imagery in stroke patients. If confirmed, this would support the clinically relevant notion that patients having greater imagery ability may be more efficient in recruiting sensory-motor areas during motor imagery exercises.

## 2. Materials and Methods

### 2.1. Participants

Eleven stroke survivors (four females) with chronic stage upper extremity hemiparesis participated in this study after giving informed consent in accordance with the Helsinki declaration. Their age ranged from 42 to 74 (60.8 years ±10.7). The mean Fugl-Meyer Total motor score for the impaired arm was 36.6 ± 8.3 (maximum motor performance score is 66 points for the upper extremity). The mean Fugl-Meyer sensory score for the impaired arm was 10 ± 3.2. The FMA is a tool that assesses reflex activity, coordination, and voluntary movement in and out of synergy patterns. Thirty-three individual items are rated on a 3-point ordinal scale (0 to 2), with a maximum possible score of 66 for upper extremity motor function; eight individual items are rated on a 3-point ordinal scale (0 to 2) with maximum possible score of 24 for sensory function. Lower score indicates a higher degree of impairment. Stroke latency ranged from 1 to 11 months before study entry (mean 6.0 ± 2.6). The average of Mini Mental State Examination (MMSE) scores for the group were 28.5 ± 1.2 (the total score for the MMSE ranges from 0 to 30). 

All volunteers reported being right-hand dominant prior to stroke, as determined by the Edinburgh Inventory [26]. Six patients had right hemiparesis, five patients had left hemiparesis. Patient demographic and clinical data are listed in Table 1.

Inclusion criteria required that participants were at least 18 years old and survived their first stroke within 24 months prior to enrolment. Participants could not be engaged in formal physical rehabilitation programs. Individuals were independent in standing, toilet transfer, and the ability to maintain balance for at least 2 minutes with arm support. Upper extremity movement criteria included the ability to actively extend their affected wrist ≥20° and extend 2 fingers and thumb at least 10° with a Motor Activity Log (MAL) score of less than 2.5 [27]. The MAL is a semistructured interview for hemiparetic stroke patients to assess the use of their paretic arm and hand, in terms of amount of use (AOU) and quality of movement (QOM), during activities of daily living; scores range from 0 to 5.

### 2.2. Assessing Imagery Ability Using the MIQ-RS

The Movement Imagery Questionnaire-Revised for Stroke (MIQ-RS) was used to investigate imagery ability of each participant [28]. The MIQ-RS is a tool for examining movement imagery ability primarily aimed at the upper extremity in able-bodied young people [28], older able-bodied, and stroke survivors [29]. This instrument assesses visual and kinaesthetic movement imagery ability and is comprised of eight items (four visual and four kinaesthetic) that reflect everyday functional movements: bending forward, pushing (an object like a door), pulling (an object like a door handle), and reaching for and grasping (an object like a drinking glass). Each item entails imagining a movement, visually or kinaesthetically and then rating the ease or difficulty of imagining it on a 7-point scale from 1 = very hard to see/feel to 7 = very easy to see/feel. The internal consistency of the MIQ-RS has been validated with Cronbach's coefficients exceeding 0.95 for both the visual and kinaesthetic subscales [29].

### 2.3. MRI Acquisition

Magnetic resonance (MR) scans were performed on a 3 Tesla Siemens Trio whole-body scanner (Siemens Medical Solutions, Malvern, PA), using a standard quadrature headcoil. Functional images with blood oxygenation level-dependent (BOLD) contrast were acquired using a T2*-weighted single-shot gradient-recalled echoplanar imaging (EPI) sequence. Axial slices of 3 mm-thickness were acquired to provide full-brain coverage (35 slices for each subject) with the following parameters: repetition time (TR) = 2350 ms, echo time (TE) = 28 ms, in-plane resolution = 3.0 × 3.0 mm, in-plane matrix = 64 × 64. A high-resolution whole-brain anatomic image was also acquired for each subject, using a 3D magnetization-prepared rapid acquisition gradient echo (MPRAGE) sequence (TR = 2300 ms, TE = 3.02 ms, inversion time = 1100 ms, flip angle = 8°), consisting of 176 sagittal slices of 1 mm-thickness (in-plane resolution = 1 × 1 mm, in-plane matrix = 256 × 256). 

The participant lay supine in the scanner with both arms outstretched close to the body. When advanced to the scan position (head centered in the magnet bore), the person's hand was located at the flared magnet aperture and was therefore unimpeded by the magnet bore in moving fingers and thumb. Foam padding wrapped around the arm and under the hand was used to minimize body movement, reduce transfer of vibration from the gradient coils, and ensure volunteer comfort. A mirror positioned above the persons' eyes provided unobstructed visualization of images projected on a screen at the magnet's rear aperture. Head restraint straps and foam blocks were utilized to minimize head movement. Sound-attenuating headphones were used to muffle scanner noise. Separate imagery and execution stimulation runs were performed.

### 2.4. fMRI Task

**Figure 1:**
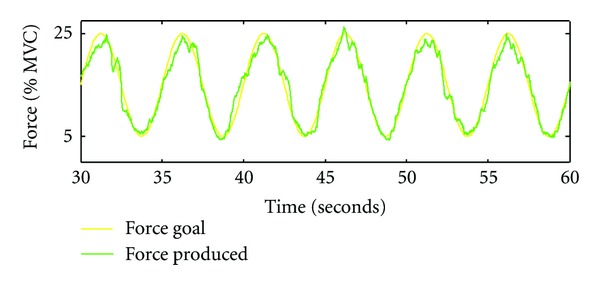
Representative force tracking of a single practice trial (i.e., one 30 second trial). The green line corresponds to subject's performance whereas the yellow line represents the target.

All patients were introduced to the task outside the scanner and allowed to practice until they were comfortable with the procedure. The task consisted of tracking a sinusoidal wave by the continuous pinching of a force transducer (Mini-40 Model, ATI Industrial Automation, Garner, N.C., U.S.A.) between the thumb and index finger of the hand using an isometric precision grip. The amplitude of the force was set between 5–25% of the individual maximum pinch force. Patients were instructed to track the sinusoidal waveform as accurately as possible, while receiving real time visual feedback (Figure 1). For the imagined task, participants were asked to track the same sinusoidal waveform while not moving their fingers. Visual feedback of both the force and sinusoidal waveform ensured that each person performed the task at the same pace during both executed and imagined movements.

A block design was used in which 30-second blocks of passive rest and 30-second blocks of the task alternated; there were three stimulation blocks per run, with rest blocks beginning and ending each run. Over the course of the experiment, each volunteer performed four different runs, counter-balanced for order between volunteers:

1. pinch affected (PA), involving active force-tracking with the affected hand;
2. imagine affected (IA), involving imagining force-tracking with the affected hand;
3. pinch unaffected (PU), involving active force-tracking with the less affected hand;
4. imagine unaffected (IU), involving imagining force-tracking with the less affected hand.

A visual textual cue (“Pinch”, “Imagine”, or “Rest”) prompted the participants to start each task. The entire scanning session lasted about one hour. To ensure that no finger movements were occurring during rest and during mental imagery task, the experimenter remained near the patients inside the scanner during each run, and the position trace was monitored continuously (i.e., checked for deviations from baseline). When performance was unacceptable, instructions were repeated to the volunteer,and the scanning run was performed again.

In order to confirm that volunteers had followed instructions during the “imagine” runs, they were asked a brief series of questions on a 5 point-Likert scale about (a) how well they performed the mental imagery tasks (1: not well to 5: very well), (b) the ease of performing the task (1: not easy to 5: very easy), (c) whether they felt sensations in the affected hand during imagery (1: not at all to 5: a lot), and (d) whether they *saw *the action during imagery (1: not at all to 5: a lot). Self-reported vividness of imagery was also assessed (1: not at all to 5: a lot), as was the presence of detail in motor images (1: not at all to 5: a lot).

### 2.5. Data Analyses

Three measures were employed to assess tracking accuracy: the relative root mean squared error (RRMSE), the time within a range (TWR) of 2.5% above and 2.5% below target force, and the coefficient of coordination (*K*_*c*_). Each measure is described more precisely in turn.

The grip force modulation task was tailored to each subject by defining the range of the target sine wave as 5%–25% of the subject's maximum pinch grip force. To account for differences in the amplitude of the target force (e.g., interpatient and intrapatient variability), and to assess the performance of the sinus task we calculated the relative root mean square error (RRMSE) between the target force *F*_*G*_ and the measured output force *F*_*p*_ over the trial time *t* [30] (1). RRMSE values close to zero suggest good control of grip force modulation:

(1) $$\begin{array}{c} \text{RRMSE}=\sqrt{\frac{1}{T}\sum_{t=2}^{T}\frac{{\left(F_{p}\left(t\right)-F_{G}\left(t\right)\right)}^{2}}{{\max\left(F_{G}\right)}^{2}}}. \end{array}$$

Time spent within a target range (TWR) of 2.5% above and 2.5% below the target sine wave was calculated for each participant in each condition. A total range of 5% was chosen because it constitutes a good safety margin when manipulating objects. When the force applied to an object is about 5% of its weight, people can manipulate it fairly well. When greater forces are applied, dexterity begins to decline. TWR was computed for each 30 second trial-block and averaged across trials. TWR values close to 30 seconds suggest a normal level of accuracy on matching the target force.

The coefficient of coordination (*K*_*c*_) between the target force and the subject-produced force describes the dynamic characteristics of pinch grip force modulation. Specifically, *K*_*c*_, as defined in (2) below, is the product of the correlation coefficient between the target signal and the force response, and the correlation coefficient for the corresponding time rates. A *K*_*c*_ value close to 1.0 suggests normal coordination of grip force. The smoothness of grip force produced is quantified by *K*_*c*_ as it describes not only the force, but its relationship to the velocity components of force. Full details of the grip force data analysis methods are described in Kurillo and colleagues [31]:

(2) $$\begin{array}{c} K_{c}=\rho \left(F_{G},F_{p}\right)\cdot \rho \left(\frac{dF_{G}}{dt},\frac{dF_{p}}{dt}\right). \end{array}$$

### 2.6. Image Processing

Image processing and analysis were performed using SPM2 (http://www.fil.ion.ucl.ac.uk/spm/). The brain images of patients with lesions located in the left brain side were flipped about the stereotactical midsagittal plane so that, for the purposes of group analysis, all the lesions were presented on the right brain side [32]. 

Individual analyses for each patient were performed. Echoplanar images were motion-corrected, adjusted for differences in slice acquisition times, and spatially warped to the standard Montreal Neurological Institute (MNI) stereotactical space using a 12-parameter affine transformation followed by nonlinear warps. The parameters defining the spatial normalization into MNI space were estimated using the subject's high-resolution T1-weighted anatomical scan, which had been previously coregistered to the mean EPI volume. EPI volumes were then spatially smoothed with a Gaussian filter (FWHM = 8 mm). Motion parameters were stored and used as nuisance variables in the generalized linear model (GLM) analysis of task-related activations. Every experimental run used a blocked design and task-related activity was modelled with a boxcar regressor convolved with a canonical hemodynamic response function. Thus a participant's estimated fMRI response for a given condition was the average response across all three blocks of that condition within the condition's run. At the group level, random-effects analyses were performed: one-sample *t*-tests and one-way ANOVAs were used to compare the four within-subject conditions [33]. We applied a threshold of *P* < 0.005 uncorrected for multiple comparison and cluster size *k* > 15 voxels.

To identify overlapping areas of activations between motor imagery and motor execution, a conjunction analysis [34] was performed using SPM2. This analysis allowed detection of brain areas that displayed a similar level of activation for the conditions “Imagery of the affected hand” and “Pinching with the affected hand”. 

Further region of interest (ROI) analyses were conducted using the SPM anatomy toolbox [35]. ROIs were selected in eight *a priori* hypothesised areas in both hemispheres, Brodmann areas 4a, 4p, 3a, 3b, 6, 1, 2, and the hippocampus. The location and extension of the anatomical ROIs were derived from the three-dimensional probabilistic cytoarchitectonic maps embedded in the SPM anatomy toolbox (http://www.fz-juelich.de/inb/inb-3/spm_anatomy_toolbox). To compensate for a small shift difference between the stereotactic spaces defined in SPM and the anatomy toolbox, a suitable translation of the coordinates origin was applied to all subjects. 

For each ROI, the average percent BOLD signal change obtained via the SPM Anatomy toolbox [35] was used as the metric of activation in the conditions of interest. Pearson's correlation analyses was performed between the percent BOLD signal change during the “imagine” task for the affected hand and the MIQ-RS visual and kinaesthetic scores for each ROI to assess correlation between individual motor imagery scores and changes in brain activation (increase or decrease of the % BOLD signal). Pearson's correlation analyses were performed using two-sided tests with a minimal level of significance set at *α* = .05 using SPSS Statistics Version 17.0 (SPSS Inc. Chicago, IL 60606).

## 3. Results

### 3.1. Behavioural Data

Age-matched healthy controls exhibit high accuracy and coordination in the task paradigm (RRMSE = 0.46; SD = 0.09; TWR = 12.16 sec; SD = 2.48; *K*_*c*_ = 0.807; SD = 0.055) [36].

 By comparison, the mean RRMSE during force tracking with the affected hand was 4.44 (SD = 5.94), the mean TWR, indicating the time the participant stayed within the range of 5% above or below the target force, was 3.28 sec (SD = 2.46), and the coordination of tracking represented by the mean *K*_*c*_ was 0.204 (SD = 0.186). These values indicate that volunteers with moderate stroke performed the motor task during the PINCH trials with more difficulty than healthy controls. The volunteers in this study exhibited no detectable force during IMAGINE trials, indicating that they remained motionless.

Data collected after each run indicate that the volunteers seemed to perform the imagery task competently. When asked about how well they were able to perform the mental imagery tasks (on a scale of 1 to 5), participants reported that, on the average, they were quite able to generate motor imagery (mean = 4; SD = 1.5) and that it was easy to perform these tasks (mean = 4.4; SD = 0.8). Moreover, patients reported a medium degree of *feeling* the affected hand in action during the imagery tasks (mean = 2.86; SD = 1.35); the degree to which they reported *seeing* the action was a bit greater (mean = 3.86; SD = 1.46). The mean score for the vividness of the generated motor images was high (mean = 4.71; SD = 0.49), as well as mean score regarding the presence of details in motor imagery (mean = 3.80; SD = 1.79).

### 3.2. Statistical Parametric Mapping Analysis of Functional Activity

To investigate the cortical substrates of motor imagery and motor execution, and their relation to one another, statistical contrasts between the active task periods (motor execution and motor imagery) and the rest conditions were performed, as well as conjunction analyses. Contrasting the active task conditions with rest revealed significant activation in the sensorimotor network for finger movements for both motor imagery (IA > Rest) and motor execution (PA > Rest).

### 3.3. Motor Imagery of Moving the Affected Hand Compared to Rest (IA > Rest)

**Figure 2:**
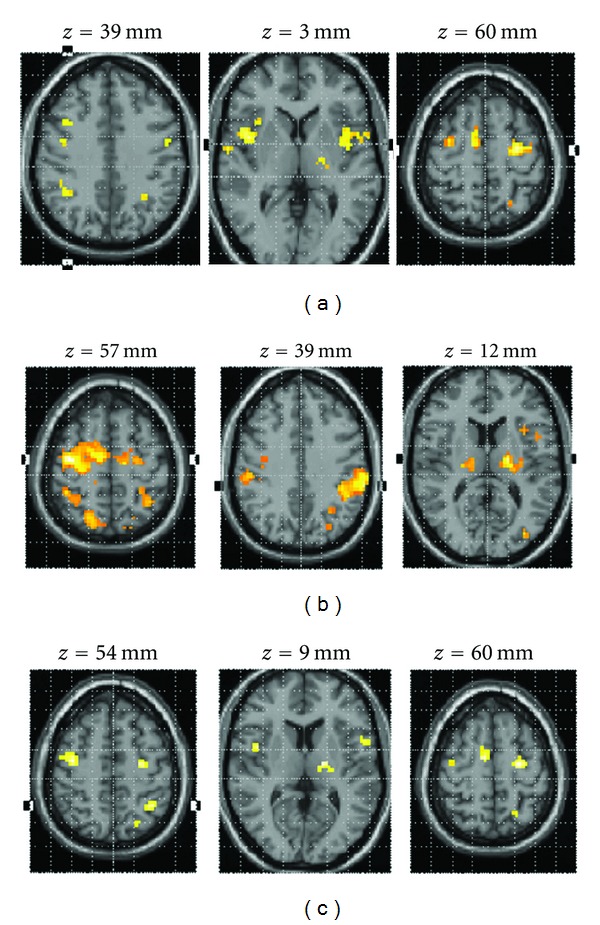
Statistical parametric maps of brain activity of different comparisons: “Imagery of the affected hand” > rest; “Pinching with the affected hand” > Rest; common areas of activations for motor imagery and motor execution. (a) Statistical parametric maps of brain activity “Imagery of the affected hand” > rest. Activations detected during imagined movement of the affected hand. (b) Statistical parametric map for the contrast pinching affected > rest. Activations detected in primary motor and somatosensory areas, as well as premotor areas and parietal lobules. (c) Common areas of activation for motor imagery and actual execution of the force tracking task with the impaired hand. Activations detected in a widely distributed frontoparietal network and subcortical structures. The SPMs threshold set at *P* < 0.005 uncorrected with a cluster size *k* ≥ 15 voxels, and superimposed on an axial slice of the MNI single-subject T1 template.

As shown in Table 2 and Figure 2(a), motor imagery of the affected hand was associated with the activation of the premotor cortex (including SMA), the insula and rolandic operculum (area BA 44), and the inferior parietal lobule of both hemispheres. In the lesioned side, we also observed an activation cluster in the pons. In the non-lesioned side, additional clusters of activation were observed in the supramarginal gyrus, the superior parietal lobule, and the thalamus.

### 3.4. Pinching with the Affected Hand Compared to Rest (PA > Rest)

Significant activations were observed in both the ipsilesional and contralesional brain sides when participants performed the tracking task with their affected hand (Figure 2(b), Table 3). In the ipsilesional hemisphere, clusters of activated voxels were observed in SMA, lateral premotor cortex (PM), primary motor cortex (M1), primary sensory cortex, supramarginal gyrus, and inferior parietal lobule. In the contralesional hemisphere, active clusters were found in the inferior and superior parietal lobule, supramarginal gyrus, and anterior intraparietal sulcus with extended activation in a portion of primary somatosensory cortex (BA 2); additional activations were observed in the insula, and inferior frontal gyrus. Bilateral activations were found in thalamus, putamen, cerebellum, inferior and middle temporal gyri, and inferior and middle occipital gyri.

### 3.5. Conjunction Analysis

As Table 4 and Figure 2(c) illustrate, a conjunction analysis revealed common activations for pinching and imagery for the affected hand. These activations occurred in the lateral premotor cortices (BA 6) of both hemispheres, and in the inferior parietal lobule, the superior parietal lobule, the inferior frontal gyrus (BA 44), the supramarginal gyrus, and the thalamus of the contralesional brain side. In the ipsilesional brain side, common areas of activation besides the lateral premotor cortex included the insula, thalamus, and the SMA.

### 3.6. Comparing Movement to Imagery of the Affected Hand (PA > IA and IA > PA)

**Figure 3:**
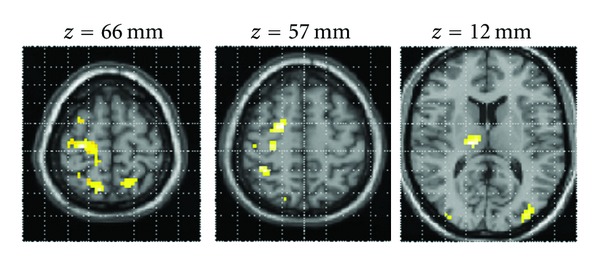
Statistical parametric map for the contrast “Pinching with the affected hand” > “Imagery of the affected hand”. The figure displays the expected activation of movement-related areas, such as primary motor and somatosensory cortex, premotor cortices, inferior, and superior parietal lobule. The map was thresholded at *P* < 0.005 uncorrected with cluster size *k* ≥ 15 voxels, and overlaid onto the MNI single-subject T1 template. There were no activation for the opposite contrast “Imagery of the affected hand” > “Pinching with the affected hand” at the threshold of *P* < 0.005 uncorrected (*k* ≥ 15 voxels).

Areas more active for pinching than for imagery included premotor cortex (BA 6), primary sensory cortex (BA 2), inferior parietal lobule, superior parietal lobule, and primary motor cortex. Significant activations also occurred in contralesional middle and inferior occipital gyrus (Table 5 and Figure 3). 

For the opposite contrast (IA > PA), no significant activations were observed at the chosen threshold combination of *P* < 0.005 and *k* > 15 voxels. However, the choice of a threshold combination more sensitive to larger clusters with lower significance peaks (*P* < 0.05 and *k* > 175) for exploratory purposes yielded significant activations in the ipsilesional precuneus, middle cingulate gyrus, superior middle temporal gyrus, and supramarginal gyrus (data not shown).

### 3.7. Activations for the Unaffected Hand

When comparing imagery of the unaffected hand to rest (IU > Rest), significant activations were observed bilaterally in the inferior parietal lobule, premotor cortices, superior frontal gyrus, supramarginal gyrus, and thalamus (Table 6). Additional activations were found in the middle frontal gyrus of the contralesional side and in the middle temporal gyrus of the ipsilesional side. Contrasting pinching versus rest for the unaffected hand (PU > rest) yielded significant activations bilaterally in inferior and superior parietal lobule, premotor cortices, supramarginal gyrus; in the ipsilesional side activations occurred in the middle primary motor cortex, primary somatosensory cortex, cerebellum, inferior and middle temporal gyrus (Table 7). In the contralesional side, significant activations occurred in the inferior frontal gyrus, hippocampus, thalamus, insula and rolandic operculum, inferior and middle occipital gyrus.

Cortical areas showing activation during pinching compared to imagery with the unaffected hand (PU > IU) included in a cluster of activations in ipsilesional primary motor and somatosensory cortices; also ipsilesional premotor cortex, rolandic operculum, and putamen showed significant activity. Significant activations occurred in contralesional primary motor and somatosensory cortices, premotor cortex, and inferior parietal lobule. The opposite contrast (IU > PU) revealed significant activation in contralesional precuneus while imagining movement when compared to execution of the same movement with the unaffected hand. 

### 3.8. Correlations between Percent BOLD Change on the Imagine Task and Imagery Ability on the MIQ-RS

The secondary aim of the study was to investigate correlations between individual brain activity during motor imagery and individual imagery for kinaesthetic and visuomotor experience. We hypothesized that individual variability in imagery ability modulates the involvement of primary motor and somatosensory cortex during motor imagery, giving rise to differential patterns of sensorimotor reenactments [11]. Brain activity was assessed during the motor imagery task for the affected hand, and related to a selected measure of imagery ability (i.e., MIQ-RS).

### 3.9. Correlations with Kinaesthetic Motor Imagery Ability

Significant correlations were found between kinaesthetic motor imagery ability and BOLD activity in somatosensory and motor areas during the motor imagery of the affected hand. Specifically, two ROIs showed significant correlations: Brodmann area 4p and Brodmann area 3a. Each correlation is addressed in turn.

**Figure 4:**
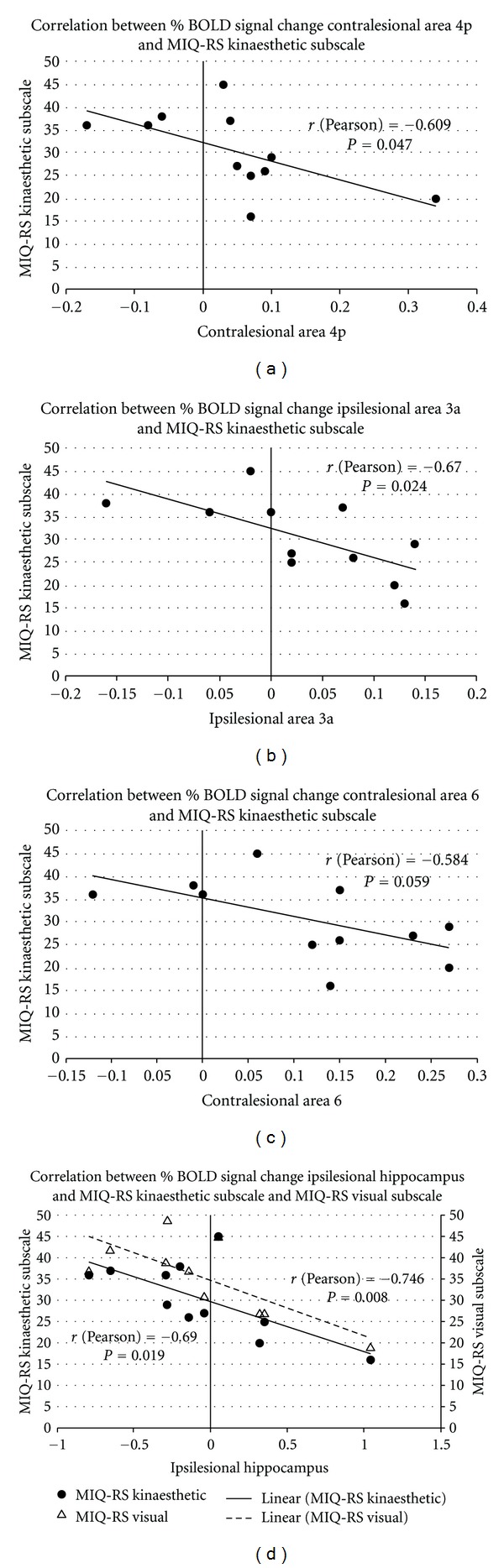
Correlation of percentage BOLD signal change in regions of interest (ROI) during motor imagery and individual MIQ-RS motor imagery ability scores. (a) and (b) show a significant correlation of the kinesthetic imagery subscale scores and the percentage BOLD signal change in areas 4p and 3a, respectively. The plots indicate that higher kinesthetic motor imagery ability correlate with a decreased (and in some cases negative) percentage BOLD signal change in contralesional primary motor cortex (area 4p) and ipsilesional primary somatosensory cortex (area 3a) during imagined actions of the affected hand. Interestingly, the percent BOLD signal change in ipsilesional hippocampus was correlated with both kinaesthetic (closed circles) and visual (open triangles) imagery subcales scores, as shown in (d). (c) show a trend of correlation between percentage BOLD signal change in contralesional area 6 and kinesthetic motor imagery ability scores.

Kinaesthetic imagery scores correlated negatively with the percent BOLD signal change in contralesional area 4p of the somatosensory system (*r* = −0.609, *P* = 0.047) (Figure 4(a)), and the ipsilesional somatosensory area 3a (*r* = −0.670, *P* = 0.024) (Figure 4(b)). In the above described correlations, patients S1, S4, S5, and S11 showed negative percentage BOLD signal changes. 

Finally, kinaesthetic imagery scores correlated negatively with the percent BOLD signal change in the ipsilesional hippocampus (*r* = −0.690, *P* = 0.019). Negative percentage BOLD signal changes have been shown in patients S1, S2, S4, S5, S6, S8, and S9 (Figure 4(d), filled circles and Table 1). 

Also we found a correlation, albeit only at a trend level and not statistically significant, between kinaesthetic imagery scores and percent of BOLD signal change in contralesional area 6 (*r* = −0.584, *P* = 0.059) (Figure 4(c)).

### 3.10. Correlations with Visual Motor Imagery Ability

A significant correlation between visual imagery ability (MIQ-RS visual imagery scores) and BOLD signal change was observed in the hippocampus of the ipsilesional hemisphere (Figure 4(d) open triangles; *r* = −0.746, *P* = 0.008). Note the negative percentage BOLD signal changes in patients S1, S2, S5, S6, S7, S8 (see Figure 4(d), open triangles, and Table 1). Visual imagery ability did not correlate significantly with BOLD activity in sensorimotor areas. Similarly, no significant correlations were found between visual imagery ability and BOLD activity in primary and secondary visual cortices.

## 4. Discussion

The results of this study support the simulation hypothesis of motor cognition, that is, of regions that are commonly activated by motor execution and motor imagery [10]. Furthermore, these shared activations appear to be preserved in patients with moderate motor impairment following stroke.

Evidence provided by the conjunction analysis showed that area BA 6, most notably ventral and dorsal lateral premotor cortex was similarly activated during motor imagery and execution in both hemispheres. This finding complements those from previous neuroimaging studies employing motor imagery in healthy participants [12, 19, 37] in which motor imagery consistently activated lateral premotor cortex. These findings are particularly relevant because evidence in stroke patients suggests that the ipsilesional premotor cortex can be functionally reorganized to manage basic parameters of movement, a function usually assigned to M1 [38]. Recent primate studies similarly suggest that functional recovery after focal lesions in M1 can be mediated by reorganized activity in ipsilesional premotor cortex [39]. Several human studies have further identified similar cortical areas of increased activation in the dorsal premotor cortex of the lesioned hemisphere in patients with chronic stroke who exhibited substantial motor recovery [40]. Therefore, the data presented in the current study provides supporting evidence that motor imagery is able to activate the premotor cortex in stroke patients suggesting a preservation of the similarities between action execution and imagery observed in healthy subjects.

The observed activation of the ipsilesional SMA during both the imagery and movement tasks is in-line with the findings of a study by Naito and colleagues [41]. When healthy subjects imagined self-controlled continuous wrist movements, activations occurred in SMA during both imagined right wrist movement and kinaesthetic illusion of wrist movements in absence of overt motor movements. Again, this finding is consistent with the present observation that SMA, a secondary motor-related area, was active during both motor imagery and motor execution, suggesting that these two processes share common mechanisms.

In our study, both contralesional inferior frontal gyrus (BA 44) and the ipsilesional insula were active during both motor imagery and motor execution. The finding that motor imagery and motor execution both activate BA 44, is reasonable, given that this area of cortex has been show to represent hand movements, not only speech [42]. Several studies indicate that BA 44, part of the so-called human mirror neuron system, is the human homologue of monkey area F5 [43], a region that is involved both in the performance and observation of actions. Using fMRI, Buccino and colleagues [44] confirmed that the observation of hand and mouth movements activates bilateral inferior frontal gyrus (area 44), and areas of the parietal lobe. Furthermore, other studies [20, 45, 46] have demonstrated that the opercular portion of the inferior frontal cortex is involved in human motor imagery. Therefore, the present findings provide further support to the hypothesis that in stroke patients, as in healthy subjects, action simulation underlies motor imagery [10].

The activation of the insula may be attributable to the kinaesthetic components of motor imagery given that the insula has been found (along with premotor areas, superior parietal lobe and somatosensory cortices) to be active during tactile imagery and perception of tactile stimuli [47]. Moreover, the insula appears to be involved in the integration of multimodal sensory signals for voluntary movements and for generation of simulated actions [48]. Therefore, our data support the hypothesis that such multimodal integration process is required in imagined movements as well as in action execution.

The results of the present study with stroke patients parallel the finding in healthy volunteers [18, 49] of another common area of activation for imagined and executed tasks in the inferior parietal lobule (IPL). The finding of IPL activation in the non-lesioned hemisphere for stroke survivors is particularly interesting, given that the IPL, as the inferior frontal gyrus, is also a component of the human mirror-neuron system [43] and has been found to be active during imagined grasping movements, action observation, and visual presentation of graspable objects [44, 50, 51]. Furthermore, IPL is also activated by action execution, for example, object manipulation [42]. The IPL may thus play a role in the integration of visual and somatosensory information during action execution as well as during motor imagery [52, 53].

The conjunction analysis in the present study revealed an activation of bilateral thalamus during both motor imagery and execution, indicating that the overlap of circuits for movement execution and imagery is not limited to the neocortex but extends subcortically in stroke patients. This is again consistent with results from imaging studies of healthy people showing activation of subcortical structures during motor imagery [18, 51].

Notably, actual execution of the finger force-tracking task in our study activated both primary motor and somatosensory areas, as well as premotor areas, parietal lobules (inferior and superior), occipital gyri (middle and inferior), and subcortical structures. In contrast, imagery-related activity was not significant in primary motor and somatosensory cortices for the affected hand.

The precuneus and the supramarginal gyrus, while not surviving the statistical threshold combination of *P* < 0.005 and *k* > 15 voxels, were however shown to be activated using a less stringent threshold of *P* < 0.05 and *k* > 175 voxels. The imagery-predominant activity found in the precuneus and supramarginal gyrus is consistent with previous evidence [18, 20] of greater activation in the parietal regions during motor imagery of finger movement compared to motor execution. The precuneus is important for motor imagery because its activation appears related to the generation of spatial information required for motor imagery tasks [54]. Also, the medial parietal cortex (including the precuneus) and the supramarginal gyrus are key nodes in the “default mode network” [55], a set of brain regions with coherent activity that has been recently proposed to be involved in acts of “self-projection” [56].

Our study demonstrates anatomofunctional similarities in brain activations between motor imagery and motor execution in stroke patients, involving a widely distributed frontoparietal network and subcortical structures. We did not find evidence from the group level analysis that motor imagery of the hemiparetic hand activates primary sensorimotor areas. The lack of evidence at a group level analysis of the involvement of M1 during motor imagery in our stroke patients is partially inconsistent with results provided by Sharma and colleagues [57]. These authors [57] show that in a sample of well-recovered subcortical stroke patients, motor imagery of the affected hand activated several cortical motor areas including M1; specifically Brodmann area 4p showed a positive correlation with motor performance. We speculate that the differences in the level of impairment could explain the different findings. As the involvement of M1 was correlated to motor outcomes, it is reasonable that our sample of less-recovered patients did not show at a group level analysis activation of M1 during motor imagery.

Activation of M1 during motor imagery is indeed controversial in the extant literature [20, 21, 58–61] and the precise function of M1 in motor imagery remains an open issue in able-bodied people. The present findings are consistent with the analysis of Hanakawa and colleagues [37] who classified brain regions according to their involvement in motor execution, planning and imagery. Hanakawa's classification of brain areas involve the type I “movement-predominant” areas, the type II areas showing similar activity between imagery and movement (e.g., frontoparietal cortical circuits), and the type III areas, showing greater imagery-related activity than motor-planning related activity (e.g., pre-SMA, frontal eye field). Type I areas are subdivided in Ia areas and Ib areas; Ia areas (e.g., primary motor cortex, primary and secondary somatosensory cortex, cerebellum) show clear movement-related activity with almost no imagery-related activity. The type Ib areas, such as dorsal premotor cortex and anterior parietal cortex, show salient movement related activity with mild imagery-related activity. The difference between type Ia and Ib areas supports the hypothesis that motor imagery is represented, in able-bodied people, in the distributed motor network characterized by a functional gradient from executable to imaginative functions. The type Ib and type II brain areas active during motor imagery in our cohort of stroke patients are consistent with the hypothesis that such areas continue to exhibit similar movement-related and imagery-related activity after stroke, as they do in healthy people [18, 20]. Our results also show that frontoparietal circuits represent an overlapping network of areas between motor imagery and execution [18, 20, 37, 58]. The present findings support the action simulation hypothesis [10, 53] in that activation of type Ib movement-related areas during action simulation is consistently weaker than during execution, whereas type Ia areas appears to be mainly devoted to motor execution.

Our observations of the neural correlates of motor imagery and execution involving the unaffected hand in stroke patients are in agreement with studies on healthy subjects. Motor imagery of the unaffected hand compared to rest condition revealed a pattern of significant activations in a frontoparietal network (bilaterally inpremotor cortex, supramarginal gyrus, inferior parietal lobule, superior frontal gyrus). This frontoparietal network has been widely acknowledged to be involved in motor cognition in healthy participants [10]. Furthermore, the overt execution of the finger-tracking task with the unaffected hand showed activations in well-established motor-related areas, as well as in premotor cortices, primary motor cortex, subcortical structures, and so forth [62]. Our data in the unaffected hand showing specific activation in the contralesional precuneus during motor imagery compared to motor execution is consistent with previous studies [18, 20].

**Individual Differences in Cognition Modulate the Degree of Similarity between Neural Substrates of Motor Imagery and Execution in Stroke Patients**

Although the group results at a population level show no activation of primary motor and somatosensory cortices during imagined movements (type Ia areas), ROI analyses showed individual differences in the activation of these areas. In these analyses, we assessed the correlation between the activation magnitudes of specific regions during motor imagery with individual differences in imagery ability. In particular, we were interested in knowing whether motor imagery ability is related to the re-activation and stimulation of primary sensory-motor areas while patients are imagining moving the affected hand [37].

The results showed that individual differences in motor imagery for this group of stroke patients were indeed correlated with neural activation in primary motor cortex and primary somatosensory cortex. However, the observed correlations between neural activity in sensory-motor areas and motor imagery were of opposite sign compared to what we hypothesized; that is, that greater kinaesthetic imagery ability would be correlated to increased activation of the relevant sensorimotor circuits. In our sample, stroke survivors with greater kinaesthetic motor imagery ability showed lower activation and in some individual cases a deactivation (negative % BOLD signal change) in contralesional primary motor cortex (area 4p) and in ipsilesional primary somatosensory cortex (area 3a) during imagined actions of the affected hand. Taken together, these data indicate that stroke patients who felt the kinaesthetic task was very easy to imagine (i.e., higher kinaesthetic subscore) exhibited lower cortical activation, and in some cases exhibited suppression of activation in these areas, compared to patients with lower kinaesthetic subscores.

The exact mechanism is unclear, however we purport that it may be necessary to actively inhibit overt execution of represented motor actions while imagining. This possible mechanism may explain the deactivation observed in areas 4p and 3a [10]. A recent study by Kasess and colleagues [63] involving healthy volunteers showed that SMA exerts a suppressive influence over the primary motor cortex during kinaesthetic motor imagery, when motor plans are being formed but movement execution is being suppressed. Our group analyses supports these findings as we showed SMA to be a shared and common area of activation for motor imagery and motor execution, while primary sensorimotor areas were not significantly activated.

Our brain-behaviour correlational data in stroke patients with moderate motor deficit is interesting in that individuals with higher kinaesthetic imagery capacity may be suppressing activation (as indicated by the negative percentage BOLD signal change) of primary sensorimotor areas. It seems reasonable to assert that in these patients with higher scores of kinaesthetic imagery ability, SMA is inhibiting sensorimotor areas while, patients with lower kinaesthetic imagery ability show greater activation in areas 4p and 3a. Other future studies beyond Kasess and colleagues [63] should further elucidate this suppressive mechanism related to different imagery ability in healthy subjects.

An alternative interpretation could be that the degree of subjectively perceived difficulty—and therefore the subjective ratings—in performing the imagery task, is mediated by the activation or deactivation of the sensorimotor areas. That is, these areas would mediate the interoceptive feeling of effort while struggling to imagine the tracking task. Then, higher activation in these areas would correspond to lower reported scores on easiness to imagine the movement. However, the former interpretation is more plausible than the latter.

Notably, findings reported by Kimberley and colleagues [25] are inconsistent with the correlations that we observed between kinaesthetic motor imagery ability and contralesional area 4p and ipsilesional area 3a. Specifically Kimberly et al. did not report significant correlations between motor imagery ability and brain activation in primary motor or somatosensory cortex in stroke patients. Differences in experimental design may explain the discrepant results. In Kimberley et al. motor imagery ability was assessed, using a modified version of Hall and Pongrac's motor imagery questionnaires [64] that does not differentiate kinaesthetic versus visual imagery ability. Furthermore, ROI definition was based on a subjective manual tracing task using non-standard structural landmarks that does not allow accurate labelling of neural components. On the other hand, the significant correlations observed in the present study are based, first, on a psychometric instrument that distinguishes kinaesthetic and visual imagery, and second, on the localization of specific primary motor and somatosensory areas by quantitatively defined probabilistic maps.

In addition, we observed significant negative correlations between both kinesthetic imagery and visual imagery ability with BOLD activations in ipsilesional hippocampus. In particular, patients with higher imagery ability scores (both visual and kinaesthetic) showed negative and lower activation in ipsilesional hippocampus whereas individual with lower imagery ability scores had positive and higher activation in the ipsilesional hippocampus. These correlations are important given the significant role of the hippocampus in spatial memory and spatial navigation [65]. Deactivation of this structure has been found for different memory tasks [66, 67]. Rekkasa and collegaues [68] showed a decreased BOLD signal in the right hippocampus with retrieval of spatial aspects. Other studies demonstrate decreased BOLD signal in association with active processing [69] where successful memory encoding typically involves not only activations but also deactivations of memory-related brain areas. These data provides evidence to supporting the notion that decreased neural activity is associated with successful cognitive performance [68, 69]. It is reasonable therefore those patients in our study with higher motor imagery ability, a process where spatial memory is essential, demonstrate deactivations (or activations close to zero) in ipsilesional hippocampus, whereas patients with lower imagery ability scores show greater activation in this area.

As expected, no significant correlations were observed between visual imagery scores and the percent BOLD signal change in sensory-motor areas. On the other hand, no correlations were found between visual imagery scores and the percent of BOLD signal change in primary and secondary visual cortices either. The absence of correlations between visual imagery scores and activity in visual cortices might be seen as incongruent with evidence reported by Cui and colleagues [70] that self-reported vividness is strongly correlated with the visual cortex activity. It is worth noting, however, that these authors assessed self-reported vividness of visual imagery, whereas we focused on self-reported ability to visually imagine movements specifically of the upper limb, which may be only partially correlated with imagery vividness. Moreover, in their visualization task participants were blindfolded, whereas in our case patients were asked to keep their eyes open during the imagery task as well as during the rest condition.

The methodological limitations of our study should be mentioned. There was a large heterogeneity of lesion location in our sample and therefore interruption of corticospinal fibers varies greatly; with it varies the associated brain damage to surrounding structures. This results unavoidably in altered brain activation patterns with very large standard deviations. Moreover all patients included in the study were right-hand dominant prior to stroke, while some of them had left or right hemiparetic as consequence of right or left lesions. In literature previous studies [71, 72] showed differences between right and left hemiparetic stroke patients in brain activations during motor tasks. Future study designs that include a wider sample of patients with similar stroke types, infarct location and severity, stroke interval as well as a comparison between right and left hemiparetic groups will further clarify our findings.

## 5. Conclusion

From a clinical perspective, these results are relevant to verify the assumption that cognitive processes such as “action simulation” engage a wide range of frontoparietal and premotor areas, due to the anatomofunctional similarities between the neural substrates of motor imagery and motor execution. The present study is relevant as it provides adjunctive data and specifications on neural network related to motor imagery in subcortical stroke patients introducing motor imagery ability as important variable. From the neuroimaging evidence presented in this study with stroke patients, the involvement of BA 4p and 3a in motor imagery seems to be differentially correlated to individual kinesthetic imagery ability. In our select group of stroke patients with mild motor deficit higher kinesthetic imagery ability appears to deactivate or activate to a lesser degree primary sensorimotor areas during motor imagery, whereas the opposite observation is seen in patients with lower kinesthetic imagery.

Controlled prospective studies, with appropriate samples sizes, specific patient inclusion/exclusion criteria, and suitable outcomes, are needed to elucidate our findings. Studies aimed at analysing neural correlates and inhibitory mechanisms in patients with defined lesion locations and healthy volunteers are necessary to substantiate our observations.

## Figures and Tables

**Table 1:** Patient demographics: age (years), gender, hemiparetic limb (affected side), Mini Mental State Exam total score (MMSE, maximum score = 30), time since stroke (months), stroke location, Fugl-Meyer upper limb total motor score (maximum score 66), scores on the MIQ-RS total scale, kinaesthetic and visual subscales scores.

Subject	Age (years)	Gender	Affected side	MMSE	Poststroke (months)	Stroke location	Fugl-Meyer	MIQ-RS	MIQ-RS	MIQ-RS
							Total motor score	Total scores	Kinesthetic scores	visual scores
1	55	Female	R	30	5	Left thalamic haemorrhage	27	75	36	39
2	55	Male	R	27	1	Left basal ganglia infarct	52	63	26	37
3	74	Female	L	30	9	Right middle cerebral artery infarct	32	35	16	19
4	65	Female	R	28	7	Left middle cerebral artery infarct	28	76	38	38
5	54	Male	L	27	11	Right putamen haemorrhage	29	73	36	37
6	50	Male	L	30	5	Right lacunar infarct (globus pallidus)	40	79	37	42
7	69	Female	L	28	8	Right middle cerebral artery infarct	34	58	27	31
8	42	Male	L	30	5	Right pontine infarct	46	78	29	49
9	55	Male	R	28	7	Left internal capsule	42	52	25	27
10	62	Male	R	28	7	Left thalamic intercerebral haemorrhage	36	47	20	27
11	73	Male	R	28	5	Left pontomedullary junction	31	90	45	45

**Table 2:** Significant clusters of activation for the main effects of “Imagery of the affected hand” (IA > Rest). Single-voxel uncorrected threshold *P* < 0.005, cluster size *k* ≥ 15 voxels. BA: Brodmann area at given coordinates.

IA > REST	Regions	BA	^ #^Voxels	*T*_peak_	MNI		
					*x*	*y*	*z*
Non-lesioned brain side	Precentral gyrus	6^12^	**179**	5.18	−60	3	30
	Insula			4.94	−39	0	6
	Inferior frontal gyrus	44^13^		4.48	−57	9	18
	Precentral gyrus	6^74^	**107**	6.97	−24	−12	57
	Supramarginal gyrus		**40**	5.31	−60	−45	27
	Superior parietal lobule		**27**	5.74	−21	−66	53
	Thalamus		**26**	4.90	−12	−15	6
	Inferior parietal lobule		**23**	3.88	−30	−51	39

Lesioned brain side	Precentral gyrus	6^10^	**280**	6.72	42	−3	48
	Rolandic operculum	44^8^		5.85	45	0	15
	Insula			5.38	45	6	6
	Supplementary motor area	6^93^	**41**	5.69	12	0	57
	Inferior parietal lobule		**34**	3.99	39	−48	39
	Pons		**30**	3.17	3	−25	−4

^#^Voxels: number of voxels in cluster. MNI: coordinates in the Montreal Neurological Institute standard brain space. *T*_peak_ = maximum *T* score in cluster. The percentage of voxels from the cluster falling within the named region is indicated by the superscripted values (obtained by the SPM Anatomy Toolbox).

**Table 3:** Significant clusters of activation for the main effects of “Pinching with the affected hand” (PA > Rest). Single-voxel uncorrected threshold *P* < 0.005, cluster size *k* ≥ 15. BA: Brodmann area at given coordinates.

PA > REST	Regions	BA	^ #^Voxels	*T*_peak_	MNI		
					*x*	*y*	*z*
Non-lesioned brain side	Inferior parietal lobule		**546**	7.10	−39	−51	48
	Supramarginal gyrus			7.40	−57	−36	36
	Superior parietal lobule			4.51	−15	−78	51
	Inferior parietal lobule	2^16 ^		4.20	−30	−42	51
	Postcentral gyrus	2		3.79	−27	−42	57
	Thalamus		**494**	7.17	−15	−12	9
	Precentral gyrus	3a^2^		5.58	−48	−6	24
	Thalamus			4.60	9	−9	0
	Putamen			4.53	−27	−12	0
	Insula			4.54	−33	12	15
	Inferior frontal gyrus (p.opercularis)			4.17	−45	9	15
	Precentral gyrus	6^2^		3.95	−54	−3	56
	Middle temporal gyrus		**176**	6.20	−51	−60	0
	Inferior temporal gyrus			5.27	−51	−57	12
	Inferior occipital gyrus			5.09	−39	−69	6
	Middle occipital gyrus			4.38	−36	−87	0
	Cerebellum (IV-V)		**38**	5.28	−12	−48	−18
	Cerebellar vermis (4-5)			3.69	0	−48	−6

Lesioned brain side	Precentral gyrus	6^53^	**782**	7.25	39	−12	57
		SMA^16^		6.58	9	−18	72
		4p^2^		3.42	39	−18	39
	Postcentral gyrus	3b^2^		3.69	42	−21	48
	Inferior parietal lobule	2^32^	**270**	5.78	39	−42	51
	Supramarginal gyrus			4.61	54	−30	48
	Middle temporal gyrus	V5^6^	**182**	7.63	54	−66	0
	Inferior temporal gyrus			4.77	57	−54	15
	Middle occipital gyrus			4.22	42	−81	3
	Inferior occipital gyrus			3.77	33	−78	9
	Superior parietal lobule		**86**	5.18	18	−75	54
	Precuneus			3.62	9	−66	66
	Amygdala		**86**	5.19	27	−12	6
	Putamen			4.17	33	−12	−3
	Thalamus			3.37	18	−24	9
	Cerebellum (IV-V)		**16**	3.63	18	−48	−15

^#^Voxels: number of voxels in cluster. MNI: coordinates in the Montreal Neurological Institute standard brain space. *T*_peak_: maximum *T* score in cluster. The percentage of voxels from the cluster falling within the named region is indicated by the superscripted values (obtained by the SPM Anatomy Toolbox).

**Table 4:** Significant clusters of activation for the conjunction analysis of the tasks' pair “Imagery of the affected hand” (IA) and “Pinching with affected hand” (PA). Single-voxel uncorrected threshold *P* < 0.005 cluster size ≥15. BA: Brodmann area at given coordinates.

Conjunction analysis IA and PA	Regions	BA	^ #^Voxels	*T*_peak_	MNI		
					*x*	*y*	*z*
Non-lesioned brain side	Inferior parietal lobule		**68**	4.45	−33	−51	51
	Precentral gyrus	6^79^	**49**	4.74	−24	−12	57
	Inferior parietal lobule		**28**	3.74	−60	−30	42
	Supramarginal gyrus			3.51	−60	−36	36
	Thalamus		**25**	4.17	−15	−12	6
	Superior parietal lobule		**23**	3.42	−21	−57	60
	Precentral gyrus	6^48^	**18**	3.50	−54	0	36
	Inferior frontal gyrus (p.opercularis)	44^79^	**17**	3.26	−57	9	15

Lesioned brain side	Precentral gyrus	6^78^	**48**	4.34	42	−9	57
	Thalamus		**39**	4.45	6	−27	0
	Insula		**36**	3.66	45	3	9
	Supplementary motor area	6^93^	**25**	4.40	9	−6	60

^#^Voxels: number of voxels in cluster. MNI: coordinates in the Montreal Neurological Institute standard brain space. *T*_peak_: maximum *T* score in cluster. The percentage of voxels from the cluster falling within the named region is indicated by the superscripted values (obtained by the SPM Anatomy Toolbox).

**Table 5:** Significant clusters of activation for the comparison between “Pinching with affected hand” (PA) and “Imagery of the affected hand”. For the comparison PA > IA, a single-voxel uncorrected threshold *P* < 0.005 and cluster size *k* ≥ 15 was chosen. BA: Brodmann area at given coordinates.

PA > IA	Regions	BA	^ #^Voxels	*T*_peak_	MNI		
					*x*	*y*	*z*
Non-lesioned brain side	Middle occipital gyrus		**182**	4.94	−39	−90	0
	Inferior occipital gyrus			4.72	−39	−81	−3
	Inferior temporal gyrus			3.26	−45	−66	−9
	Postcentral gyrus		**30**	4.21	−57	−21	33
	Inferior parietal lobule	2^15^		3.07	−51	−27	42
	Precuneus		**26**	3.72	−15	−54	63
	Superior parietal lobule			3.34	−21	−54	66
	Paracentral lobule	4a^40^ 4a(les)^15^	**17**	4.84	−3	−36	75

	Precentral gyurs	6^42^		4.73	27	−21	66
	Inferior parietal lobule	2^20^		3.83	36	−42	51
	Superior parietal lobule	2		3.74	36	−45	57
	Postcentral gyrus	4p^2^		3.56	15	−33	63
	Supramarginal gyrus		**244**	3.37	66	−24	30
	Paracentral lobule	6		3.31	9	−21	72
	Precentral gyrus	6		3.23	42	−15	63
Lesioned brain side	Postcentral gyrus	3b^6^		2.95	45	−21	48
	Postcentral gyrus	1^2^		3.15	60	−21	48
	Superior frontal gyrus		**56**	4.02	21	−3	57
	Superior parietal lobule		**43**	4.20	12	−63	66
	Thalamus		**40**	4.21	18	−18	12
	Parahippocampal gyrus		**35**	3.73	18	−12	−21
	Middle occipital gyrus		**17**	3.09	39	−87	15
	Calcarine gyrus	17^99^	**16**	3.36	15	−96	3

^#^Voxels: number of voxels in cluster. MNI: coordinates in the Montreal Neurological Institute standard brain space. *T*_peak_: maximum *T* score in cluster. The percentage of voxels from the cluster falling within the named region is indicated by the superscripted values (obtained by the SPM Anatomy Toolbox).

**Table 6:** Significant clusters of activation for the main effects of “Imagery of the unaffected hand” (IU > Rest). Single-voxel uncorrected threshold *P* < 0.005, cluster size *k* ≥ 15. BA: Brodmann area at given coordinates.

IU > REST	Regions	BA	^ #^Voxels	*T*_peak_	MNI		
					*x*	*y*	*z*
Non-lesioned brain side	Inferior parietal lobule		**527**	9.86	−45	−42	51
	Supramarginal gyrus			5.74	−57	−24	33
	Precentral gyrus	6^54^	**301**	6.19	−36	−18	60
	Middle frontal gyrus			5.26	−27	6	48
	Thalamus		**68**	5.48	−21	−21	21

Lesioned brain side	Supramarginal gyrus		**155**	6.43	54	−30	45
	Inferior parietal lobule	2^27^		4.36	42	−42	45
	Thalamus		**36**	5.71	21	−27	15
	Middle temporal gyrus		**27**	4.79	63	−39	9
	Precentral gyrus	6^18^	**21**	4.16	54	−6	42
	Precentral gyrus	6^93^	**18**	5.40	42	−9	54

Bilateral	Supplementary motor area	6^49^	**212**	6.06	6	−6	69
	Superior frontal gyrus	6^46^		4.50	−18	−6	69

^#^Voxels: number of voxels in cluster. MNI: coordinates in the Montreal Neurological Institute standard brain space. *T*_peak_: maximum *T* score in cluster. The percentage of voxels from the cluster falling within the named region is indicated by the superscripted values (obtained by the SPM Anatomy Toolbox).

**Table 7:** Significant clusters of activation for the main effects of “Pinching with the unaffected hand” (PU > Rest). Single-voxel uncorrected threshold *P* < 0.005, cluster size *k* ≥ 15. BA: Brodmann area at given coordinates.

PU > REST	Regions	BA	^ #^Voxels	*T*_peak_	MNI		
					*x*	*y*	*z*
Non-lesioned brain side	Precentral gyrus	6^7^	**918**	7.26	−57	0	33
	Inferior frontal gyrus (p. opercularis)			6.89	−45	9	12
	Hippocampus			5.81	−21	−27	−6
	Insula			5.08	−33	15	12
	Thalamus			5.01	−12	−18	9
	Rolandic opercolum			4.76	−57	0	6
	Middle occipital gyrus	17^8^	**376**	7.73	−36	−84	0
	Inferior temporal gyrus			5.82	−51	−66	−9
	Inferior occipital gyrus			3.72	30	−75	−6
	Calcarine gyrus	18^55^ 17^18^	**18**	4.33	3	−87	9

Lesioned brain side	Inferior temporal gyrus		**1196**	9.27	51	−69	−3
	Middle temporal gyrus			7.56	45	−66	0
	Middle occipital gyrus			7.55	39	−81	0
	Cerebellum			5.25	24	−51	−18
	Superior parietal lobule		**992**	8.44	15	−72	54
	Inferior parietal lobule			8.06	45	−52	54
	Superior occipital gyrus			8.05	27	−75	33
	Precentral gyrus	4p^5^		6.44	33	−21	45
	Postcentral gyrus	2^21^		4.63	48	−27	42
	Supramarginal gyrus			4.60	60	−33	39

Bilateral	C. inferior parietal lobule	2^8^	**3181**	9.13	−45	−30	45
	I. supplementary motor area	6^16^		8.24	15	−9	63
	I. middle cingulate cortex			7.63	9	6	45
	C. superior parietal lobule			7.62	−24	−60	57
	C. supra marginal gyrus			7.61	−60	−27	42
	C. inferior parietal lobule			7.52	−36	−51	54
	I. supplementary motor area	6		7.38	3	0	51
	C. precentral gyrus	6^17^		7.06	−21	−15	69

^#^Voxels: number of voxels in cluster. MNI: coordinates in the Montreal Neurological Institute standard brain space. *T*_peak_: maximum *T* score in cluster. The percentage of voxels from the cluster falling within the named region is indicated by the superscripted values (obtained by the SPM Anatomy Toolbox). I: ipsilesional; C: contralesional.
